# Evaluation of the positive predictive value of a rapid Immunochromatographic test to detect Campylobacter in stools

**DOI:** 10.1186/1757-4749-4-17

**Published:** 2012-12-04

**Authors:** Pauline Floch, Julien Goret, Emilie Bessède, Philippe Lehours, Francis Mégraud

**Affiliations:** 1 Université de Bordeaux, French National Reference Center for Campylobacters, 33076, Bordeaux, France; 2 Laboratoire de Bactériologie, Hôpital Pellegrin, Place Amélie Raba-Léon, 33076 Bordeaux cedex, Bordeaux, France

**Keywords:** Culture, ELISA, PCR, Specificity, Near-patient test

## Abstract

The recently developed rapid immunochromatographic tests (ICT) have the potential to provide a quick and easy diagnosis of Campylobacter enteritis in comparison to culture. In a previous study we found them sensitive but lacking in specificity. The aim of the present study was to focus on the problem of specificity and determine the positive predictive value (PPV) of a positive result of the ImmunoCard Stat! Campy (Meridian Bioscience, Cincinnati, OH, USA). For this purpose, the stools positive by ICT were cultured according to 3 different protocols: Karmali agar, Preston enrichment broth subcultured on Karmali agar, and a filtration method on a blood agar without antibiotics, all incubated for 7 days at 37°C. Out of 609 stools from adults and children with community acquired enteritis, the reference methods detected 25 positive cases (4.1%) (culture: 19, specific PCR and ELISA both positive: 6) and the ICT: 31 including the 25 true positives. The PPV was 80.6%. We conclude that ICT is a good method to screen Campylobacter positive stools but because of its lack of specificity the positive stools must be tested by another method.

## 

Campylobacters, especially *Campylobacter jejuni* and *Campylobacter coli*, are the main cause of bacterial enteric infections worldwide [1]. These infections can also lead to extraintestinal localizations and severe long-term complications, e.g. Guillain Barré Syndrome [2]. The main diagnostic method used is currently culture, which is considered to be technically demanding and culture may underestimate the real incidence of this infection if the special culture needs are not completely fulfilled. Other techniques have been developed, e.g. molecular methods (real-time PCR), and ELISAs which have a better sensitivity than culture and appear to be specific [3]. They give a result in a few hours but are also technically demanding. Recently, immunochromatographic tests which allow to obtain a result within a few minutes and are very easy to perform, have been developed. The first studies performed have shown a good sensitivity but apparent lack in specificity. Our aim was to evaluate the positive predictive value (PPV) of such tests in comparison to a reference.

During a 3-month period (August-October 2011) an immunochromatographic test (ImmunoCard STAT!Campy, Meridian Bioscience, Cincinnati, OH, USA) was applied on all the stools received at the bacteriology laboratory of our teaching hospital from patients (adults and children) with community-acquired enteric infection. In case of positive result, culture was performed by using 3 different methods: Karmali agar (Oxoid, Dardilly, France), overnight enrichment in Preston broth followed by culture on Karmali agar, and blood agar after a filtration step on a 0.65 μM filter (Merck Millipore, Billerica, MA, USA). The media were incubated for 7 days at 37°C in a microaerobic atmosphere (gas pack in jars) and observed daily. The colonies suspected to be *Campylobacter* species were confirmed on morphology, oxidase activity and formally identified by MALDI-TOF mass spectrometry [4]. The remaining stools were then frozen at −80°C to perform ELISA and PCR at the same time. After thawing, an ELISA (Ridascreen Campylobacter, r-Biopharm AG, Darmstadt, Germany) was performed according to the suppliers’ recommendations. DNA was extracted from stools (NorDiag, Arrow, Helsinki, Norway) and an in-house real-time PCR based on the fluorescence resonance energy transfer (FRET) principle, targeting the *gyr*A gene and specific for *C. jejuni* and *C. coli* was carried out. After amplification, a melting curve analysis allowed to differentiate *C. jejuni* and *C. coli *[5]. A stool was considered positive for Campylobacter if culture was positive or in case of negative culture if both the ELISA and the real-time PCR were positive.

Out of the 609 stools tested, the ImmunoCard STAT!Campy was positive in 31 cases (5.1%). Culture was positive in 19 cases (3.1%) (18 *C. jejuni*, 1 *C. coli*) and all were detected by the rapid test. The enrichment method and the filtration method did not allow detection of more positive stools than Karmali agar. The ELISA and the specific real-time PCR detected all the cases positive by culture as well as six other cases. There was a perfect agreement between these 2 additional methods. The 6 extra cases were all *C. jejuni* according to the PCR results. According to our gold standard, 25 stools (4.1%) were considered positive for Campylobacters, all detected by the rapid test but 6 cases were false positive giving a positive predictive value of 80.6% (Table 1). The characteristics of the 25 patients are presented on Table 2. The same percentage was isolated from adults and children (<16y).

**Table 1:** Distribution of the positivity profiles of the cases using the different techniques for detection of Campylobacters

**ImmunoCard STAT! Campy®**	**Culture**	**Ridascreen Campylobacter®**	**Real-time PCR**	**Total**
+	+	+	+	19
+	-	+	+	6
+	-	-	-	6

**Table 2:** Distribution of the 25 positivity cases studied according to age group and gender

**Age group**	**Total number**	**Gender**	
		**Male**	**Female**
Infants	1	1	0
Children	11	6	5
Adults	13	1	12
**Total**	**25**	**8**	**17**

During this period, there were 3 other culture positive cases by standard culture which could not be included in the protocol.

As in a previous study [3], we used as gold standard a culture positive and in case of negative culture 2 methods based on different principles (ELISA and PCR) which strengthen the reality of the result.

In this study, Campylobacter culture performed according to 3 different protocols, and incubating the plates during a week, did not allow to improve the results, the standard Karmali medium was indeed positive within 1 to 2 days except in one case.

It is interesting to note that the combination of ELISA and real-time PCR was always positive when culture was positive, but allowed to detect 6 other cases giving a sensitivity of 76% for the culture in this context. There was a perfect correlation between ELISA and PCR.

The ImmunoCard STAT!Campy did not appear to lack sensitivity since it detected the 19 cases positive by culture, and also an additional 6 cases positive by ELISA and PCR. In contrast, it lacks specificity since there were 6 false positive cases which lead to a PPV of 80.6%, so insufficient to be a stand-alone diagnostic test.

However, the possibility still exists that the false positive detected are true positive, if we are in the eventuality of a method more sensitive than the current reference methods, but it remains to be proven. Indeed, we hypothesized that *Campylobacter* species other that *C. jejuni*/*C. coli* cross reacting antigenically with them, but not growing on Karmali agar, could be present. Culture after filtration did not allow confirmation of this hypothesis. Another possibility would be to look to raised Campylobacter antibodies in blood which we did not have the opportunity to carry out in this study.

An advantage of this test is its convenience and rapidity compared to the other methods. Given that it appears sensitive but lacks specificity, a possibility would be to use it to screen stools for Campylobacters and to use another method for confirmation. Applying this strategy in a cohort such as in this study would have saved a large number of cultures.

A cost benefit analysis is warranted to confirm the interest of this strategy.

## Competing interests

The authors declare that they have no competing interests.

## Authors’ contributions

PF and JG performed the tests, gathered all the information and wrote a first draft of the manuscript. PL, EB & FM designed the study, analyzed the data and corrected the manuscript. All authors read and approved the final manuscript.
